# Development, Phenotypic Characterization and Genomic Analysis of a *Francisella tularensis* Panel for Tularemia Vaccine Testing

**DOI:** 10.3389/fmicb.2021.725776

**Published:** 2021-08-11

**Authors:** Beth A. Bachert, Joshua B. Richardson, Kevin D. Mlynek, Christopher P. Klimko, Ronald G. Toothman, David P. Fetterer, Andrea E. Luquette, Kitty Chase, Jessica L. Storrs, Ashley K. Rogers, Christopher K. Cote, David A. Rozak, Joel A. Bozue

**Affiliations:** ^1^Division of Bacteriology, United States Army Medical Research Institute of Infectious Diseases, Frederick, MD, United States; ^2^Center for Genome Sciences, United States Army Medical Research Institute of Infectious Diseases, Frederick, MD, United States; ^3^Division of Biostatistics, United States Army Medical Research Institute of Infectious Diseases, Frederick, MD, United States; ^4^Biodefense Reference Material Repository, United States Army Medical Research Institute of Infectious Diseases, Frederick, MD, United States

**Keywords:** *Francisella tularensis*, vaccine, tularemia, *Francisella* pathogenicity island, virulence, mouse, animal modeling

## Abstract

*Francisella tularensis* is one of several biothreat agents for which a licensed vaccine is needed to protect against this pathogen. To aid in the development of a vaccine protective against pneumonic tularemia, we generated and characterized a panel of *F. tularensis* isolates that can be used as challenge strains to assess vaccine efficacy. Our panel consists of both historical and contemporary isolates derived from clinical and environmental sources, including human, tick, and rabbit isolates. Whole genome sequencing was performed to assess the genetic diversity in comparison to the reference genome *F. tularensis* Schu S4. Average nucleotide identity analysis showed >99% genomic similarity across the strains in our panel, and pan-genome analysis revealed a core genome of 1,707 genes, and an accessory genome of 233 genes. Three of the strains in our panel, FRAN254 (tick-derived), FRAN255 (a type B strain), and FRAN256 (a human isolate) exhibited variation from the other strains. Moreover, we identified several unique mutations within the *Francisella* Pathogenicity Island across multiple strains in our panel, revealing unexpected diversity in this region. Notably, FRAN031 (Scherm) completely lacked the second pathogenicity island but retained virulence in mice. In contrast, FRAN037 (Coll) was attenuated in a murine pneumonic tularemia model and had mutations in *pdpB* and *iglA* which likely led to attenuation. All of the strains, except FRAN037, retained full virulence, indicating their effectiveness as challenge strains for future vaccine testing. Overall, we provide a well-characterized panel of virulent *F. tularensis* strains that can be utilized in ongoing efforts to develop an effective vaccine against pneumonic tularemia to ensure protection is achieved across a range *F. tularensis* strains.

## Introduction

*Francisella tularensis* is a gram-negative bacterium that causes the potentially life threatening and debilitating disease tularemia. *F. tularensis* is able to infect a wide range of animal species, including humans. *F. tularensis* can be transmitted to humans through a number of routes, the most common being the bite of an infected insect or other arthropod vector (Jellison, 1950; Markowitz et al., 1985; Ellis et al., 2002; Goethert et al., 2004). Human illness can range from the ulceroglandular form (most common and naturally occurring form of the disease) to more serious pneumonic or typhoidal tularemia (Ellis et al., 2002). In pneumonic tularemia (of most concern for the biodefense community), infection progresses from the lungs to other organs, primarily the liver and spleen (Saslaw et al., 1961a; Evans et al., 1985; Dennis et al., 2001; Twenhafel et al., 2009; Fritz et al., 2014; Heine et al., 2016). The risk of infection is associated mainly with two subspecies, the more virulent *F. tularensis* subsp. *tularensis* (type A) and the less virulent *F. tularensis* subsp. *holarctica* (type B).

Until agreement of the Biological Weapons Convention, *F. tularensis* had been weaponized for the potential use by several countries (Dennis et al., 2001). Due to its high pathogenicity, low infectious dose, and demonstrable virulence after aerosolization, *F. tularensis* poses a serious potential threat and therefore is classified by the United States (U.S.) Department of Health and Human Services as a Tier 1 Select Agent (Saslaw et al., 1961a, b; Harris, 1992; Dennis et al., 2001). A medical countermeasure gap for the U.S. military and biodefense communities is the lack of a Food and Drug Administration (FDA) approved vaccine to prevent pneumonic tularemia.

A live vaccine strain (LVS) derived from a type B strain of *F. tularensis* was previously developed and used in the former Soviet Union (Eigelsbach and Downs, 1961; Tigertt, 1962). In the U.S., LVS has been administered as an Investigational New Drug status vaccine to at risk laboratory workers since it does provide some level of protection against respiratory challenge with *F. tularensis* in human volunteers (McCrumb, 1961; Saslaw et al., 1961a). However, LVS is not an ideal vaccine and has disadvantages, including the potential for systemic side effects and lack of complete understanding of the basis of attenuation (McCrumb, 1961; Saslaw et al., 1961a; Rohmer et al., 2006). In addition, obtaining approval for LVS is complicated by its unknown history, instability of colony morphology, and potential reversion to virulence.

Thus, new efforts are underway to derive better pneumonic tularemia vaccine candidates for biothreat concerns which could gain FDA approval. The incidence of pneumonic tularemia in the U.S. and worldwide is very low and geographically spread out which would not be conducive to conducting human clinical vaccine trials. Therefore, animal models would be needed to test the protective efficacy of future tularemia vaccines. Two models, which have been shown to be similar to pneumonic tularemia in humans, are the rat aerosol challenge model (Jemski, 1981; Ray et al., 2010; Hutt et al., 2017) and the non-human primate (specifically the cynomolgus macaque) aerosolized *F. tularensis* challenge model (Glynn et al., 2015; Guina et al., 2018).

Several prospective tularemia vaccine candidates are being pursued, including *F. holarctica* and *F. tularensis* Schu S4 attenuated variants containing deletions in *capB*, a putative capsule biosynthesis gene (Conlan et al., 2010; Jia et al., 2010, 2016), *purMCD*, involved in nutrient metabolism (Pechous et al., 2008), and *clpB*, a heat shock gene (Conlan et al., 2010; Golovliov et al., 2013). The vast majority of vaccine candidates for tularemia have only been assessed for protection against the Schu S4 isolate. However, infection with the prototypical Schu S4 strain may not represent infection with diverse *F. tularensis* strains. Twine et al. (2006) initially compared the virulence of Schu S4 (obtained from the *Francisella* Strain collection) against another type A isolate, FSC033, showing that Schu S4 was less virulent during intradermal and aerosol infection of mice (Twine et al., 2006). The type A isolates can be further divided into subpopulations A1a, A1b, and A2, using molecular typing methods. These subpopulations differ in their geographical associations and degrees of virulence. For example, type A1 isolates primarily occur in the central U.S. while the type A2 isolates are mostly found in the western U.S. (Farlow et al., 2005), and type A1b isolates have been identified to result in significantly higher mortality during human infections than any other subtype (Kugeler et al., 2009). A more recent study showed that Schu S4 (BEI # NR-643), designated as type A1a, actually exhibited decreased virulence in mice compared to other type A1a strains as well as type A1b and A2 isolates (Molins et al., 2014). However, genetic differences have been demonstrated between the source of isolates of Schu S4 leading to variation in virulence (Lovchik et al., 2021).

A successful tularemia vaccine needs to protect against a wide variety of *F. tularensis* strains, which may differ in geographic origin or virulence attributes. In order to facilitate the efficacy testing of new vaccines against pneumonic tularemia, we developed a well-characterized panel of *F. tularensis* strains currently derived from various regions of the U.S. This panel represents a variety of historical, clinical, and environmental isolates that we have characterized phenotypically and genotypically and will be essential in the development of medical countermeasures against pneumonic tularemia for future vaccine testing in the appropriate animal models.

## Materials and Methods

### Bacterial Strains

All *F. tularensis* strains used in this study are listed in Table 1. Single-use cultures were prepared by inoculating enriched chocolate agar plates obtained from Remel^TM^ with source material and incubating for 24 h at 35°C in 5% CO_2_. Bacterial growth was re-suspended in Trypticase Soy Broth (BBL^TM^) with 12.5% glycerol and stored at –70°C until needed. Single use vials from each lot were randomly checked for purity by observing colony morphologies on chocolate agar after 24, 48, and 72 h at 35°C in 5% CO_2_. The randomly selected aliquots were also Gram-stained to confirm appropriate staining and morphology under a microscope. Where indicated, *F. tularensis* was grown in Chamberlain’s Defined Medium (CDM) (Chamberlain, 1965) or brain heart infusion (BHI) broth supplemented with 1% Isovitalex (Becton Dickinson). All isolates were prepared under International Organization for Standardization (ISO) 17025 and ISO 17034 standard and are being retained and distributed via the Biodefense Reference Material Repository for this and any vaccine, therapeutic, and diagnostic studies.

**TABLE 1:** *Francisella tularensis* strains used in this study.

Strain	BRMR#	Type	Clinical description	GenBank #	Source/Reference
Schu S4	FRAN249	A1	Human ulcer, OH, 1941	CP073129	1958 USAMRIID Repository (Eigelsbach et al., 1951)
Schu S4	FRAN244	A1	Human ulcer, OH, 1941	CP073128	BEI Resources (NR-10492) (Eigelsbach et al., 1951)
Scherm	FRAN031	A1	Human, digital lesion, OH, 1944	CP073127	1954 USAMRIID Repository (Downs et al., 1947a)
Coll	FRAN037	A1	Human, digital ulcer, OH, 1945	CP073126	1948 USAMRIID Repository (Downs et al., 1947a)
2015321842	FRAN255	B	Human, male, pleura, KY, 2015	CP073125	MDH
2015315990	FRAN256	A2	Human, male, CSF, MT, 2015	CP073124	MDH
2014313438	FRAN250	A1a	Human, male bronchoalveolar lavage, IL 2014	CP073123	MDH
2017317779	FRAN251	A1a	Human, male, lung tissue, MN, 2017	CP073122	MDH
2016320786	FRAN253	A1a	Rabbit, male, spleen, MN, 2016	CP073121	MDH
2017314593	FRAN254	A1a	Tick, female, MN, 2017	CP073120	MDH

### Genome Sequencing

DNA was isolated using the QIAgen EZ1 platform. DNA for each strain was sequenced using either the PacBio Sequel, using version 2 SMRT cells and template prep kit, or the Illumina MiSeq, using the v3 600 cycle kit and Nextera Flex library prep kit. If only PacBio reads were available, reads were quality filtered (quality >0.7) and assembled using HGAP4 (Chin et al., 2013), with default assembly parameters, except the Aggressive option was set to “On.” Reads were assembled into two contigs, and manually joined in Geneious, if necessary. Illumina reads had adapters removed and were quality trimmed using Trimmomatic v.0.33 (Bolger et al., 2014). PacBio reads were corrected using the Illumina reads and the program Filtlong (Wick, 2020). PacBio and Illumina reads were *de novo* hybrid assembled using Spades (Bankevich et al., 2012) and Unicycler (Wick et al., 2017). If complete *de novo* assemblies were not generated by Spades and/or Unicycler, draft assemblies were merged using flye’s subassemblies setting (Kolmogorov et al., 2019), and manual adjustments and circularization was then done in Ugene (Okonechnikov et al., 2012) and Mauve (Darling et al., 2010). Reads were mapped back to the draft assembly with Minimap2 (Li, 2018), and errors were corrected using Pilon (Walker et al., 2014). Genbank accession numbers are included in Table 1.

### Pan-Genome Analysis

Assembled genomes were annotated using the PROKKA (Seemann, 2014) pipeline through the Galaxy platform (Afgan et al., 2018). Average Nucleotide Identity was calculated using the method of Goris (Goris et al., 2007), on the Enve-omics platform (Rodriguez-R and Konstantinidis, 2016). A pan-genome analysis was performed with ROARY (Page et al., 2015), using the ten strains in the *Francisella* panel and the reference strain Schu S4, and with paralog splitting disabled. Core genes were defined as genes present in all ten strains, while the shell and cloud genes were defined as genes present in two or more strains, and genes present in 1 strain, respectively. The shell and cloud genes constitute the accessory genome. Results were visualized using Phandango (Hadfield et al., 2017), and a principal component analysis of the presence absence table was performed in R (R. C. Team, 2020) using the micropan package (Snipen and Liland, 2015) and custom scripts. UpSet analysis was performed with the UpSetR package in R (Conway et al., 2017).

### Growth Curves

Growth assays were performed in BHI or CDM, as indicated. Assays were performed using an Infinite M200 Pro (Tecan) microplate reader in 96-well microtiter plates at 37°C with shaking. The OD_600_ was measured every 60 min. For all assays, *F. tularensis* strains were grown for 24 h on a chocolate agar plate and then resuspended in broth medium to an approximately equal OD_600_ (0.025). All samples were performed in quadruplicate and included medium controls to confirm sterility and for use as blanks to calculate the absorbance of the cultures.

### LPS Analysis

Whole-cell extracts were collected for LPS analysis from plate grown *F. tularensis* strains. Cultures were prepared at approximately equal colony forming unit (CFU) concentrations in PBS, lysed in gel loading buffer solution, and boiled for at least 45 min and confirmed to be inactivated. Samples were fractioned on NuPage Novex 4–12% Bis-Tris gels. For western analysis, fractionated material was transferred onto a nitrocellulouse membrane using an iBlot Gel Transfer Device. After transfer, the membranes were blocked with 1% skim milk in Tris Buffered Saline + Tween 20. Samples were blotted with mouse monoclonal anti-LPS antibody (F6070-02X; US Biological; Salem, MA, United States) or anti-capsule antibody (11B7; Apicella et al., 2010) at dilutions of 1:500. The loading control antibody used for all analyses was rabbit polyclonal anti-*Escherichia coli* GroEL (dilution of 1:2,000) (Enzo Life Sciences; Farmingdale, NY, United States). Bands were visualized using 3,3′,5,5′-Tetramethylbenzidine Membrane Peroxidase substrate (Kirkegaard & Perry Laboratories, Inc; Gaithersburg, MD, United States).

### Intracellular Growth Analysis

J774A.1 cells, a murine macrophage-like cell line obtained from the American Type Culture Collection (TIB-67^TM^), were seeded (∼2.5 × 10^5^ cells/well) into 24-well plates and cultured 2 days (37°C, 5% CO_2_) at which time the cells had formed confluent monolayers. The cells were maintained in Dulbecco’s Modified Eagle’s medium containing 10% heat-inactivated fetal bovine serum (Corning 10-013-CV). For the intracellular assays, *F. tularensis* was suspended in phosphate buffered saline (PBS) from a 24 h plate, and then diluted 1:5 in tissue culture medium. The bacterial suspension was added to the macrophages in 200 μl of medium to achieve a multiplicity of infection (MOI) of ∼100:1, and the MOI was confirmed from this suspension by serial dilutions and plating on chocolate agar plates. The bacteria and macrophages were allowed to co-incubate for 2 h at 37^*o*^C with 5% CO_2_. Next, the medium containing the extracellular bacteria was aspirated and replaced with fresh tissue culture medium supplemented with 25 μg/ml of gentamycin for an additional 2 h. After this incubation, samples from the tissue culture wells were washed three times with PBS. The monolayer was then lysed with 200 μl of sterile water, immediately scraped, and suspended in 800 μl of PBS. The suspension was serially diluted in PBS and plated onto chocolate agar plates. The remaining tissue culture wells were assayed for CFU recovery at the 24 h post-infection time point as described above.

### Mouse Challenges

Virulence of each of the *F. tularensis* strains was assessed in BALB/c mice (7–9 week-old obtained from Charles River Laboratories) by intranasal challenge as previously described (Bachert et al., 2019; Biot et al., 2020). Briefly, the titer of the challenge doses were determined by serial dilutions in PBS and plating on chocolate agar. Mice were anesthetized with 150 μl of ketamine, acepromazine, and xylazine injected intraperitoneally. The mice were then challenged by intranasal instillation with 50 μl from serial dilutions of *F. tularensis* suspended in PBS to an OD_600_ of approximately 0.5 from freshly swabbed plate cultures grown for 24 h. Mice were monitored several times each day for 14 days for clinical signs, and mortality rates were recorded. Humane endpoints were used, and mice were euthanized when moribund according to an endpoint score sheet.

### Statistics

Growth analysis of bacterial strains in broth media was analyzed as previously described (Zweitering et al., 1990). A logistic growth equation was used to fit the data as a function of maximum density. For mouse challenge experiments, LD_50_ values were determined under a probit model and median time to death or euthanasia (TTD) was estimated by the Kaplan–Meier method. Comparisons of the median TTD between strains are obtained from a log-normal accelerated failure time model of the form Log(TTD) = m^∗^Log(Dose) + b, where m and b are strain-specific slope and intercept terms. A Wald test was used to compare the model estimated median TTD at the middle of the dose curve. Analysis was implemented in PROC LIFEREG and PROC PROBIT, SAS version 9.4 (SAS Institute Inc., Cary, NC, United States).

## Results

### Strain Collection

The Department of Defense’s Biodefense Reference Material Repository (BRMR), which is housed at the United States Army Medical Research Institute for Infectious Diseases (USAMRIID), contains distinct *F. tularensis* isolates, which were obtained from historical and contemporary environmental and clinical sources and prepared according ISO standards and guidelines for biological reference materials. These isolates were down-selected to the 10 potential candidate strains that were used in this study (Table 1) by loosely following guidelines laid out by Van Zandt et al. (2012) to support the creation of a similarly motivated *Burkholderia* challenge panel. Most importantly, we preferentially selected strains that were known or believed to be close to the environmental or clinical source and were accompanied by clinical or laboratory evidence of virulence in humans or mammals. These strains can be broadly separated into historic and contemporary representatives of the pathogenic bacterium.

Three of the 10 strains in our panel (FRAN031/Scherm, FRAN037/Coll, and FRAN249/Schu S4) were derived from lyophilized samples (see Table 1), not long after being isolated from human patients in 1944, 1945, and 1941, respectively. The strains were isolated from human ulcers and a digital lesion (Hesselbrock and Foshay, 1945; Buchele and Downs, 1949; Eigelsbach et al., 1951). These isolates were the subject of early virulence studies in mice (Downs and Woodward, 1949) and embryonated eggs (Downs et al., 1947a), which showed that they were among the more virulent *F. tularensis* isolates.

Six of the strains used in this study (FRAN250-FRAN251 and FRAN253-FRAN256) had been isolated from clinical and environmental samples collected in the central U.S. during the past 5 years and donated to the BRMR by the Minnesota Department of Health (MDH). Four of the MDH strains came from human clinical samples, one was propagated from a rabbit spleen, and the sixth was extracted from a tick (Table 1). We deliberately included a type B strain, which had been derived from a human tissue sample, to ensure that both of the major *F. tularensis* subtypes were included in our panel. Molecular sub-typing based on SNPs as described previously (Pandya et al., 2009), showed all of the remaining MDH strains were type A1a, except for FRAN256, which was type A2. Each of the MDH-supplied strains had undergone limited laboratory manipulation before being accessioned into the BRMR and used in this study. The MDH strains had not been tested in animal virulence studies prior to now.

The remaining two isolates used for this study, FRAN244 and FRAN249, are both versions of the commonly used Schu S4 strain. FRAN244 was prepared from the BEI Resources Schu S4 isolate (NR-10492), which is commonly used in *F. tularensis* studies. We selected this widely studied Schu S4 isolate, despite its uncertain propagation history, to compare to FRAN249, which we had propagated directly from a 1958 lyophilized sample. Since Schu S4 was originally propagated from a single colony pick in 1951 (Eigelsbach et al., 1951), the lyophilized sample would have been no more than 7 years removed from the original isolate. It is worth noting that the 1958 sample, from which FRAN249 was derived, had a subpopulation with the *corC* gene containing a frameshift mutation. The *corC* gene had been previously shown to be involved in polyamine responsiveness and have a role in virulence (Russo et al., 2011). For this study, FRAN249 was propagated from a single colony pick that did not contain the *corC* mutation.

### Genomic Diversity and Pan-Genome Analysis of *F. tularensis* Panel

**FIGURE 1:**
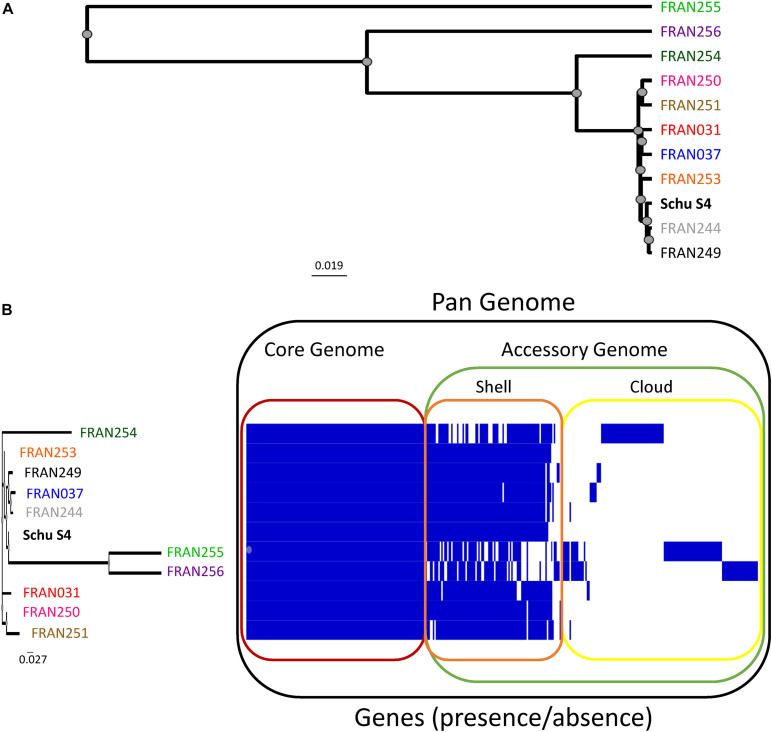
Genomic diversity and pan-genome analysis of the selected *F. tularensis* panel. **(A)** A distance tree was generated based upon the average nucleotide identity calculated for the entire genomes of our 10 strain panel and the original Schu S4 sequence (Larsson et al., 2005). The tree indicates clustering of sample matrix by unweighted pair-group method with arithmetic mean (UPGMA). **(B)** Presence/absence of all genes were assessed across the 11 genomes using Roary, and results were visualized using Phandango. Blue bars indicate the presence or absence of a specific gene across the panel, with genes shared across all strains outlined in red (core genome), genes conserved among most strains outlined in orange (shell), and poorly conserved genes outlined in yellow (cloud). The shell and cloud genes make up the accessory genome (green). The dendrogram based upon the accessory genome of strains in this panel is shown to the left. *F. tularensis* panel strains are color-coded here and in subsequent figures as follows: FRAN249 - black, FRAN244 - gray, FRAN031 - red, FRAN037 - blue, FRAN255 - green, FRAN256 - purple, FRAN250 - pink, FRAN251 - brown, FRAN253 - orange, FRAN254 - dark green.

Whole genome sequencing was performed to assess the genetic diversity of the *F. tularensis* panel. To provide a measure of nucleotide level diversity across the panel for the entire genome, we calculated the pairwise Average Nucleotide Identity (ANI), and constructed a distance tree based on this analysis (Figure 1A). On average, strains in the panel differ by approximately 0.001954%, translating to an average of 3,551 nucleotide differences between strains. The number of pairwise differences ranges from 23 (for closely related FRAN244 and FRAN249 strains) to 13,401 (for distantly related FRAN254 and FRAN255 strains). Supplementary Figure 1 shows the distance matrix for all strains. The level of diversity observed in this panel is typical of *F. tularensis*, as described previously and discussed further below (Vogler et al., 2009). In addition, we constructed a phylogeny of *F. tularensis* subsp. *holarctica*, *tularensis*, and *mediasiatica* strains that have complete genomes, including the newly sequenced strains in our panel, indicated in blue (Supplementary Figure 2). The tree is based on 100 conserved genes found within each strain. The genomes fall within three main clades corresponding to *holarctica*, *tularensis* type A2 and *tularensis* type A1. Within clade A1, the panel strains, indicated in blue, occur throughout the clade, suggesting we have captured a reasonable amount of diversity. The tree also indicates low diversity in general within the type A1 clade, similar to what we observe in our nucleotide identity analysis.

We next performed a pan-genome analysis, in order to determine the number of “core” or conserved/essential genes as compared to the unique or “accessory” genes across these strains. Figure 1B shows the pan-genome of all 10 strains of the panel and the original Schu S4 genetic sequence (Larsson et al., 2005). The core genome, defined as genes present in all strains at >95% similarity at the amino acid level, consisted of 1,707 genes (red outline). The accessory genome, outlined in green, contains the shell and core genes that are conserved at various frequencies across the panel. One-hundred and seven genes were identified in the shell genome (orange outline), conserved in two or more strains, and 126 genes were identified in the cloud genome (yellow outline), present in 1 strain. The full list of each gene in the pan-genome, along with locus tags, are listed in Supplementary Datasheet 1. A dendrogram based upon the differences in presence/absence of genes in the accessory genome is shown in Figure 1B (left panel), and mirrors the pattern observed in Figure 1A, generated from the whole genome. FRAN255 and FRAN256 cluster separately from the other strains, harboring more genes that are either unique or mutated, as compared to the rest of the panel. Similarly, FRAN254, the only tick isolate in our panel, clusters separately and contains a distinct pattern of unique genes (discussed below).

**FIGURE 2:**
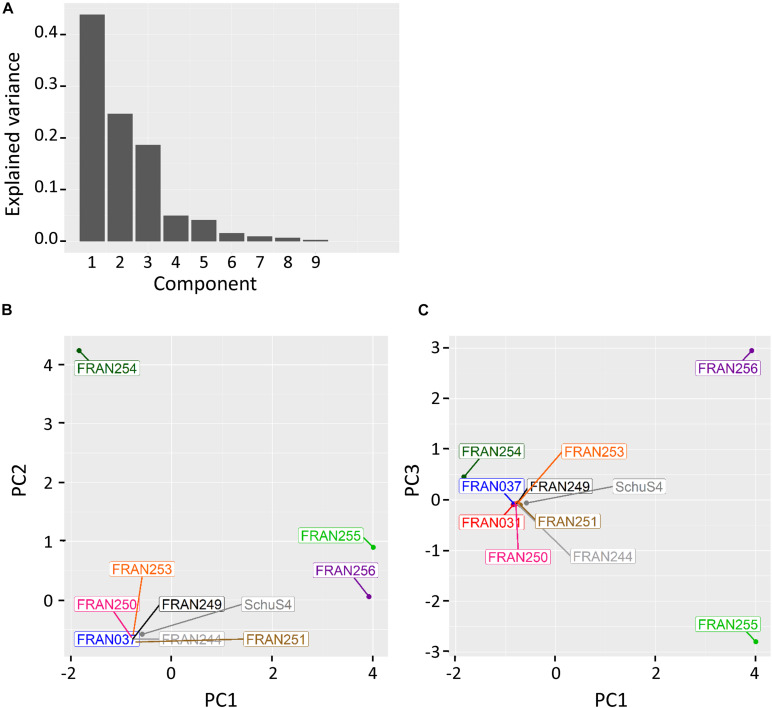
Principal component analysis of whole genome data. Prokka-annotated genomes were analyzed with Roary to identify orthologous sequences. Principal component analysis was then performed on the cloud genes using the presence/absence tables generated from the Roary analysis. **(A)** Explained variance for each principle component. PCA plots were generated for PC2 vs. PC1 **(B)** and PC3 vs. PC1 **(C)**.

In order to further assess the genomic diversity of the strains in our panel, we performed principal component analysis (PCA) using the micropan package in R. Figure 2 shows PCA plots based on cloud genes presence/absence identified by the Roary analysis. Principal components 1, 2, and 3 accounted for approximately 45, 25, and 20% of the variances across the *F. tularensis* panel, respectively (Figure 2A). PCA plots of our *F. tularensis* panel and the originally sequenced Schu S4 showed eight of the strains formed a single cluster, while the three remaining strains, FRAN254, FRAN255, and FRAN256, were isolated from the rest of the strains and from each other (Figures 2B,C). Both FRAN254 and FRAN256 are type A isolates derived from tick and male cerebrospinal fluid, respectively, while FRAN255 is a type B isolate from a male pleural sample. Collectively, the ANI, pan-genome, and PCA analyses demonstrate that the majority of the strains in our panel, including Schu S4 and its variants, have high genetic similarity, while FRAN254, FRAN255, and FRAN256 are more distant from these strains and from each other.

### Analysis of Mutated Genes Found in the *F. tularensis* Panel

**FIGURE 3:**
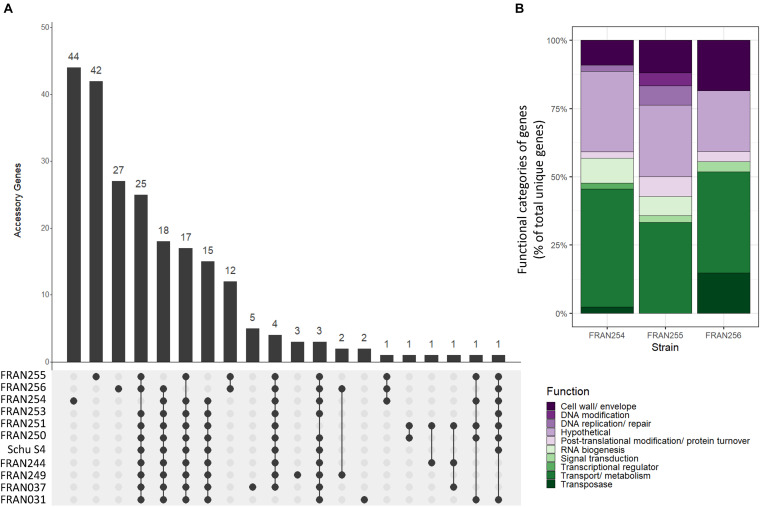
UpSet plots based on gene conservation across the *F. tularensis* panel. **(A)** UpSet plots show the intersections of gene groups among all strains. Strains are indicated in the rows, while intersections are shown in the columns. Filled circles indicate the presence of a specific subset of genes within that strain while empty circles indicate absence of the gene set within a strain. The bar graph above shows the size of each gene group intersection. For example, the first bar shows 44 genes that are unique in FRAN254, since the only filled circle corresponds to FRAN254; however, the fourth bar indicates a set of 25 genes that are conserved in all strains with the exception of FRAN254. **(B)** Stacked bar chart showing gene groups unique to FRAN254, FRAN255, and FRAN256, with colored bars indicating the functional categories assigned to each gene as a percent of the total genes.

To better understand the genetic changes driving the variation seen in the pan-genome analysis, UpSet plots were generated based on the accessory genome content of the panel strains. In the UpSet plot shown in Figure 3, strains are indicated in rows and the matrix (gray shaded region) shows the intersections, with filled circles indicating a set of genes is present in that strain, and empty circles indicating the set of genes is absent in that strain. Gene presence is defined as having greater than 95% similarity at the protein level. The corresponding bar graph shows how large these intersections are, i.e., how many genes are shared across this specific set of strains. The first 20 intersections representing the accessory genome are shown in Figure 3; the 1,707 genes within the core genome are not shown. A large number of these intersections encompass gene sets that were either unique in the FRAN254/255/256 trio or absent in these strains compared to the other *F. tularensis* strains (Figure 3). Notably, FRAN254, FRAN255, and FRAN256, which showed the most variation in the PCA analysis, had the highest number of unique gene sets, 44, 42, and 27, respectively. Twelve additional genes were shared by FRAN255 and FRAN256, while one gene, encoding a glycosyltransferase, was shared between all three strains. A total of 74 genes were absent in one or more of the trio in comparison to the rest of the panel. The remaining intersections contained 1–5 genes in each group that varied across the strains in our panel.

Data from our UpSet plot showed a large number of genes that are mutated in the FRAN254, FRAN255, and FRAN256 strains compared to the rest of our panel. We next aimed to characterize the function of these genes and the types of mutations present. BLAST search and multiple sequence alignments were carried out for each individual gene, and functional categories were assigned to each gene. Table 2 shows the frequency of mutations identified in these strains and the functions of the genes in which these mutations were found. Of the 113 mutated genes, the vast majority fell into three categories: transport (38.9%), hypothetical (24.8%), and cell wall (12.4%). The inset in Figure 3B shows a stacked bar chart of these unique genes in FRAN254, FRAN255, and FRAN256, color-coded to indicate the percentage of genes belonging to each functional category. The percentage of genes in these categories were similar across all three isolates. Differences observed in FRAN254, FRAN255, and FRAN256 consisted of 70 insertions/deletions causing frameshifts, 19 in-frame insertions/deletions, 18 unique genes, 3 missense mutations, 2 gene duplications, and a single gene fusion. The complete list of genes and corresponding mutations are detailed in Supplementary Datasheet 2. Of note, FRAN256 harbored two transposases, ISFtu1 and ISFtu2, which differed in copy number as compared to the rest of the panel. 26 copies of ISFtu2 were observed in FRAN256, while this element was repeated up to 16 times in the remaining strains. ISFtu1, however, was duplicated only nine times in FRAN256 compared to 47 copies observed in most strains. Interestingly, we also observed several unexpected mutations within the *Francisella* Pathogenicity Island (FPI) of multiple strains within our panel, described below.

**TABLE 2:** Frequency of mutations and their functional categories in FRAN254, FRAN255, and FRAN256.

	FRAN254		FRAN255		FRAN256		Total	
Functional category*	No.	%	No.	%	No.	%	No.	%
Cell wall	4	9.1	5	11.9	5	18.5	14	12.4
DNA	1	2.3	5	11.9	0	0.0	6	5.3
PTM	1	2.3	3	7.1	3	11.1	7	6.2
RNA biogenesis	4	9.1	3	7.1	0	0.0	7	6.2
Regulation	1	2.3	1	2.4	1	3.7	3	2.7
Transport	19	43.2	15	35.7	10	37.0	44	38.9
Transposase	1	2.3	0	0.0	2	7.4	4	3.5
Hypothetical	13	29.5	10	23.8	6	22.2	28	24.8
Total	44	100.0	42	100.0	27	100.0	113	100.0

*Mutated genes were assigned to one of the following functional categories: cell wall, DNA replication/repair (DNA), post-translational modification (PTM), RNA biogenesis, regulation, transport, transposase, or hypothetical. Number (No.) and frequency (%) of each functional category within strains or in total gene number are shown.

### Sequencing of the FPI Reveals Diverse Patterns of Mutations Across the *F. tularensis* Panel

**FIGURE 4:**
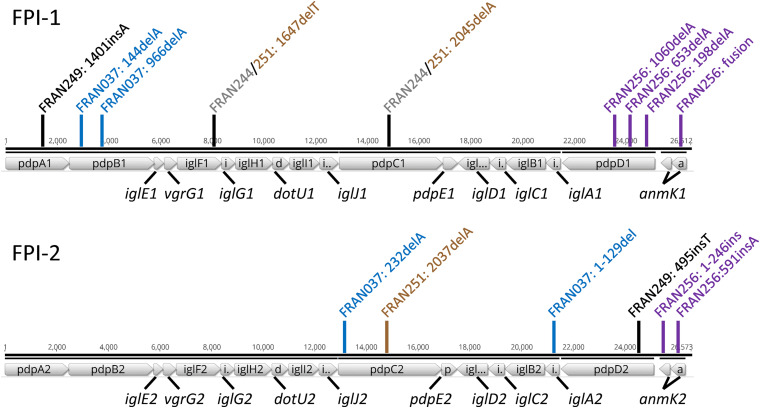
Map of mutations identified in the *Francisella* pathogenicity islands. The regions spanning *pdpA*- *anmK* from FPI-1 **(top)** and FPI-2 **(bottom)** of the Schu S4 strain are shown to scale (FTT_1344.FTT_1361c; FTT_1699.FTT_1717). Markers indicate approximate locations of mutations identified in the FPI genes, colored according to the *F. tularensis* strains in which they were observed; FRAN249 - black, FRAN244 - gray, FRAN037 - blue, FRAN256 - purple, FRAN251 - brown.

Sequencing of our *F. tularensis* panel revealed diverse mutations across both FPI in several strains. These mutations occurred in the *pdpA*, *pdpB*, *iglF*, *pdpC*, and *pdpD* genes of the first FPI, and in the *pdpC*, *iglA*, and *pdpD* genes of the second FPI (Table 3). Notably, FRAN031 completely lacked the first FPI and had an intact copy of the second FPI with no notable mutations in any of the genes. All of the mutations, with the exception of an in-frame deletion in *iglA2*, were single base-pair insertions or deletions that resulted in a frameshift and premature stop codon in each gene. A map of the mutations identified in FPI-1 and FPI-2 of the strains in our *F. tularensis* panel is shown in Figure 4. FRAN249 harbored mutations in *pdpA1* and *pdpD2*. Interestingly, FRAN244 and FRAN251 harbored identical mutations in *iglF1* and *pdpC1*, with FRAN251 containing an additional mutation in *pdpC2*. FRAN251 was the only strain in our panel harboring mutations in both copies of an FPI gene. FRAN037 contained frameshift mutations in *pdpB1* and *pdpC2*, and a unique in-frame deletion of the first 129 bp of the *iglA2* gene. Lastly, FRAN256 harbored multiple single bp deletions in *pdpD1*, presumably resulting in several truncated protein products, as well as unique *anmK* variants in both FPI regions. The *anmK* gene was represented by a single ORF in FPI-1, while *anmK1* was extended and *anmK2* was truncated in FPI-2. The diversity of FPI mutations within the *F. tularensis* panel was unexpected, although experiments described below suggest the majority of these mutations did not diminish the virulence of these strains, possibly because of the redundancy that exists between the duplicated FPIs.

**TABLE 3:** Mutations identified in the *Francisella* pathogenicity islands.

	FPI-1						FPI-2				
	*pdpA1	*pdpB1	*iglF1	*pdpC1*	*pdpD1*	*anmK*	*pdpC2*	*iglA2	*pdpD2*	*anmK2*	*anmK1*
FRAN249	1401insA								495insT		
FRAN244			1647delT	2045delA							
FRAN031	Absent	Absent	Absent	Absent	Absent	Absent					
FRAN037		144delA; 966delA					232delA	1-129del			
FRAN256					198delA; 653delA; 1060delA	Fusion				1-246ins	591insA
FRAN251			1647delT	2045delA			2037delA				

*Genes required for type VI secretion.

### Growth of the *F. tularensis* Panel in BHI and CDM

**FIGURE 5:**
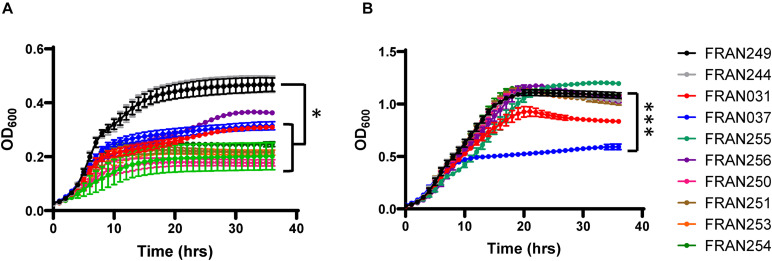
Growth assays in BHI broth and CDM. The 10 *F. tularensis* strains were grown in BHI containing 1% Isovitalex **(A)** or CDM **(B)** at 37°C, and growth was monitored by optical density. OD_600_ measurements were based upon quadruplicate samples and the experiment was repeated three times. The graphs show the average of these three experiments. Error bars represent standard error of the mean. ^∗^*P* < 0.05 for FRAN249 and FRAN244 compared to all other strains with the exception of FRAN256 **(A)**, and ^∗∗∗^*P* < 0.001 for FRAN249 and FRAN244 compared to FRAN037 **(B)**.

For the 10 strains considered for the panel, growth of the strains was assayed on both solid agar medium and in two different types of broth media. For nine of the strains, no difference in colony size was noted. However, FRAN255 formed smaller colonies when compared to Schu S4 and required longer growth time (3 days versus 2 days) to form CFU that were countable (Supplementary Figure 3). Growth of the strains was also examined in broth media to include BHI with 1% Isovitalex (complex medium) (Figure 5A) and CDM (nutritionally defined medium) (Figure 5B). When grown in BHI, the two versions of Schu S4 (FRAN244 and FRAN249) grew to a maximum OD_600_ of approximately 0.5 in agreement with our previous observations with Schu S4 (Kijek et al., 2019). In contrast, all of the other *F. tularensis* strains were never able to reach the OD_600_ observed for Schu S4 (Figure 5A). FRAN256 exhibited a consistently lower OD_600_, although not statistically significant, while the remaining seven strains reached a maximal OD_600_ that was significantly lower than that of Schu S4. However, when the strains were grown in CDM, a much higher OD_600_ was reached for all strains. The only strain from this panel which was found to be significantly decreased in maximal OD_600_ as compared to the Schu S4 strains was the FRAN037/Coll (*P* < 0.001) (Figure 5B).

### *F. tularensis* Panel Strains Show Similar Levels of Intracellular Replication in Macrophages

**FIGURE 6:**
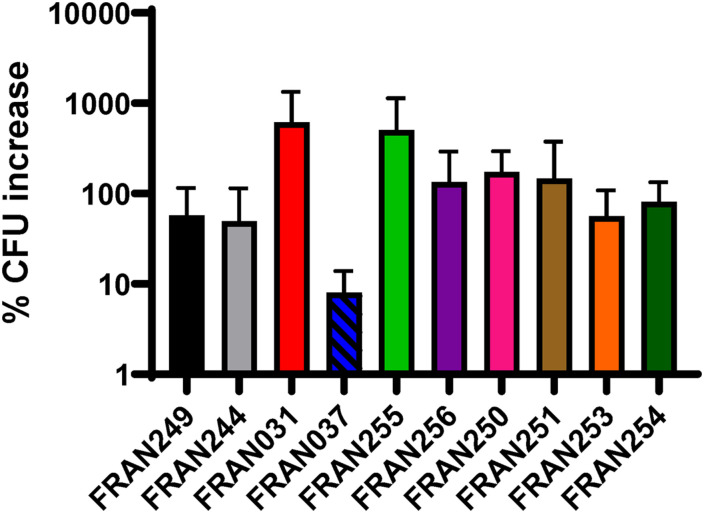
Intracellular replication of *F. tularensis* strains in J774.1 macrophages. Gentamicin protection assays were utilized to assess replication of *F. tularensis* strains in J774.1 murine macrophages. Macrophage monolayers were infected with an MOI of 100 and cells were lysed and plated to enumerate CFU at 4 and 24 h post-infection. The percent CFU increase from the 4 h to 24 h time point was calculated. No statistical significance in percent increase was observed via *t*-test.

As the ability to replicate within host cells is a hallmark of *F. tularensis* pathogenesis, we analyzed the ability of the 10 strains to grow intracellularly within J774 cells. As shown in Figure 6, all strains showed the ability to grow within the macrophage-like cells as the Schu S4 strains and increased in CFU numbers 2–3 logs, except for the FRAN037/Coll strain. Although the replication defect was not statistically significant, the results with FRAN037 suggested that this historical strain could be attenuated.

### All *F. tularensis* Isolates, Except FRAN037, Exhibited Similar Virulence in Mice

**FIGURE 7:**
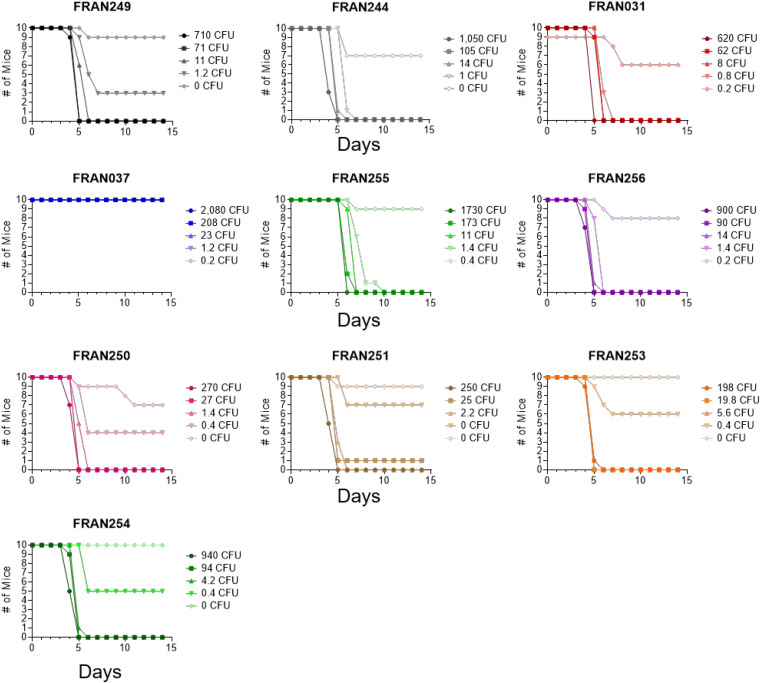
Survival data of mice challenged intranasally with *F. tularensis*. Groups of BALB/c mice (*n* = 10) were challenged and survival monitored following infection by intranasal instillation. Each group was challenged with the designated strain from the diversity panel. The calculated LD_50_ values from these experiments are included in Table 4.

To confirm the strains entered into the diversity panel were virulent, a pneumonic tularemia murine model was used. Though mice are not ideal for tularemia vaccine models, they are suitable for assessing *F. tularensis* virulence (Conlan et al., 2003; Chen et al., 2005; Fritz et al., 2014). Therefore, LD_50_ analysis was performed with all 10 strains by intranasal challenge of BALB/c mice (Figure 7 and Table 4). As expected for both versions of Schu S4 (FRAN244 and FRAN249), and in agreement with previous results from our laboratory and others (Chance et al., 2017), the LD_50_ measurements for both versions were found to be <1 CFU.

**TABLE 4:** Mouse intranasal LD_50_ and TTD measurements.

		Time to death (days)					
Strain	LD_50_	10^–2^-10^–1^	10^–1^-10^0^	10^0^-10^1^	10^1^-10^2^	10^2^-10^3^	10^3^-10^4^
	(CFU)	CFU	CFU	CFU	CFU	CFU	CFU
FRAN249	<1	N/A	>14	6.5	6	5	5
FRAN244	<1	N/A	>14	6	5	5	4
FRAN031	<1	>14	6	6	6	5	N/A
FRAN037	>2,080	N/A	>14	>14	>14	>14	>14
FRAN255*	<1	N/A	>14	8	7	6	6
FRAN256	<1	N/A	>14	6	5	5	5
FRAN250	<1	>14	6	5.5	5	5	N/A
FRAN251	<1	>14	>14	5	5	4.5	N/A
FRAN253	<1	>14	>14	5	5	5	N/A
FRAN254	<1	>14	>14	5	5	4.5	N/A

*Significantly higher TTD observed compared to other strains.

Two additional historical *F. tularensis* strains considered for the panel were FRAN031 (Scherm) and FRAN037 (Coll). The mouse LD_50_ values for these strains was originally reported as the dilution of a standard suspension which killed 50% of the mice by intraperitoneal challenge and assayed to be 10^–9.2^ and 10^–8.8^, respectively (Downs et al., 1947b). To confirm the virulence of these historical strains and obtain the LD_50_ with an actual CFU value, we tested these strains by intranasal challenge in this model. The LD_50_ value for FRAN031 (Scherm) was also found to be <1 CFU. In contrast, no mice succumbed to challenge with the FRAN037 (Coll) strain at any of the challenged doses; thus, the LD_50_ would be >2,080 CFU. Based upon the results described here, we will not be advancing FRAN037 (Coll) to be part of the diversity panel.

As no additional data were available for the clinical and environmental isolates obtained from the MDH other than listed in Table 1, we chose to confirm virulence in the mouse model prior to including these strains into the diversity panel. All proposed strains were found to be highly lethal in mice, and the LD_50_ determined to be <1 CFU.

The only virulent *F. tularensis* strain that showed any significant difference in TTD across all tested challenge doses was FRAN255 when compared to the other lethal strains. The *P*-values when comparing TTD of FRAN255 to the other virulent strains was calculated to be as follows: FRAN249 (*P* = 0.0281), FRAN244 (*P* ≤ 0.0001), FRAN031 (*P* = 0.0008), FRAN250 (*P* ≤ 0.0001), FRAN251 (*P* = 0.0004), FRAN252 (*P* = 0.0005), FRAN253 (*P* = 0.0007), FRAN254 (*P* = 0.0051), and FRAN256 (*P* = 0.0001). However, as described above, FRAN255 also displayed a slower growth on agar plates compared to all the other strains in our panel (Supplementary Figure 3) and in BHI broth when compared to most of the other strains (Figure 5A).

### Analysis of O-Antigen Profiles Across the *F. tularensis* Panel

**FIGURE 8:**
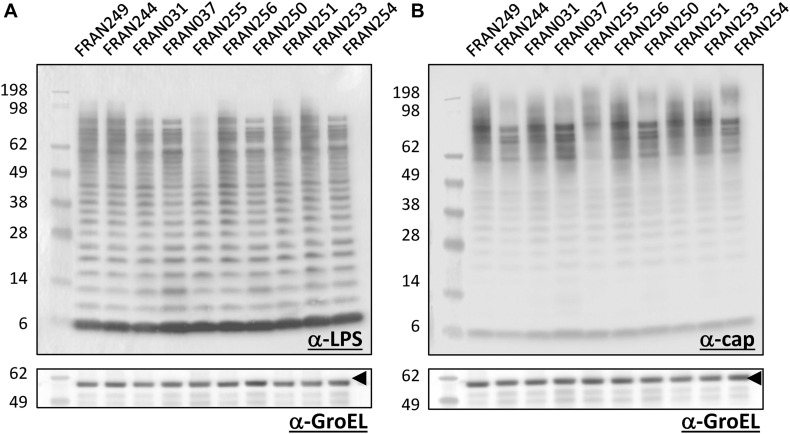
Western blot analysis of *F. tularensis* strains. Pellets of the 10 *F. tularensis* strains were lysed. Extracts were run on SDS-PAGE gels at equal concentrations and blotted with either a monoclonal antibody to the O-antigen of LPS **(A)** or capsule **(B)** of *F. tularensis*. Equal loading of sample material was demonstrated when blotting the extracts with a polyclonal antibody directed against the GroEL protein (indicated by the arrowheads). Molecular masses are indicated on the left in kDa.

One of the major virulence factors of *Francisella* is the lipopolysaccharide (LPS) and O-antigen capsule, which plays an important role in evasion of the host immune response (Sandstrom et al., 1992; Wang et al., 2006, 2007; Weiss et al., 2007; Kanistanon et al., 2008). In addition, mutations in *F. tularensis* LPS or capsule biosynthesis genes lead to attenuation (Raynaud et al., 2007; Apicella et al., 2010; Kim et al., 2012; Jones et al., 2014; Rasmussen et al., 2014, 2015; Chance et al., 2017). Therefore, we screened the O-antigen profile of our *F. tularensis* strains with monoclonal antibodies to both structures to determine if any differences were noted amongst the panel. As shown in Figure 8, when using an anti-LPS or anti-capsule monoclonal antibody for western gels, the overall banding pattern was similar for all 10 strains. A subtle difference was noted for FRAN255 in the staining intensity of the bands at the higher molecular weight region. However, the loading control for this study was anti-GroEL, and no differences in banding intensities were noted between any of the strains.

## Discussion

### Need for a Tularemia Vaccine-Testing Panel

The lack of an FDA approved tularemia vaccine is a gap for the biodefense community. LVS has been utilized as an investigational vaccine since the 1960’s. In addition, it does not provide full protection against aerosol exposure to *F. tularensis* and retains residual virulence (Eigelsbach and Downs, 1961; Tigertt, 1962). Vaccine candidates worthy of advanced development must be safer than LVS and provide comparable or greater protection against challenge with aerosolized *F. tularensis* Schu S4 (Jia and Horwitz, 2018). Significant efforts have been made toward vaccine development and characterization. Several live attenuated mutants of *F. tularensis* have been derived and tested for protective efficacy against Schu S4, including the Schu S4 deletion mutants Δ*clpB*, Δ*FTT0198/*Δ*capB* (Conlan et al., 2010) and *purMCD* (Pechous et al., 2008). A series of studies by Jia et al. (2010) assessed the efficacy of LVS Δ*capB*, containing a deletion in the putative capsule biosynthesis gene, as well as LVS Δ*capB* overexpressing the type VI secretion genes *iglA/B/C* (Jia et al., 2013, 2016, 2018). Another study has also shown that a *F. novicida iglD* mutant was able to protect both rats and NHPs against *F. tularensis* challenge (Chu et al., 2014). From both a safety and regulatory approval perspective, a subunit vaccine may be preferred to avoid the concerns of a live *Francisella* strain being used (Post et al., 2017; Mansour et al., 2018; Marshall et al., 2018; Whelan et al., 2018). However, live attenuated vaccine strains may provide better protection as they have the ability to establish a mild infection in the host, mimicking the infection of fully virulent strains and presenting appropriate antigens to the immune system to induce durable antibody and cell-mediated immune responses (Drabner and Guzman, 2001).

Regardless of the type of vaccine, the majority of tularemia protection studies typically use the prototypical Schu S4 strain for challenge. However, differences in virulence exist between different laboratory stocks of Schu S4, as shown by a recent study that used a pneumonic tularemia model with both Fischer 344 rats and New Zealand white rabbits (Lovchik et al., 2021). Ideally, a common set of index *F. tularensis* strains would be used by the biodefense community for future vaccine and therapeutic countermeasure development research to protect against pneumonic tularemia. Therefore, we generated and characterized a panel of *F. tularensis* strains for the purpose of assessing future vaccine efficacy across a range of isolates in appropriate animal model, such as aerosol challenged Fisher rats and cynomolgus macaques. With this panel, a thorough genomic, *in vitro*, and *in vivo* characterization was performed with the 10 strains. The genomic analysis consisted of whole genome sequencing using a mixture of long and short read technologies to generate complete, circularized genomes. Assembled genomes were subjected to ANI, phylogenetic and pan-genome analysis to measure the genetic variation present across the strain panel. Our *in vitro* characterization included growth curves, LPS profiles, and intracellular replication macrophage assays. We assessed initial virulence of the panel as measured by LD_50_ determinations using an intranasal murine model of infection. However, as mice are extremely susceptible to *F. tularensis*, ongoing studies are currently assessing any virulence differences between the panel strains in the Fischer 344 rat aerosol challenge model (Jemski, 1981; Ray et al., 2010; Hutt et al., 2017).

### Genomic Diversity of the *F. tularensis* Panel

Whole genome sequencing of our *F. tularensis* panel revealed high genetic similarity within *F. tularensis* (>99% nucleotide identity). We identified an average of 3,551 nucleotide differences among strains, with a range of 23 (closely related strains) to 13,401 (distantly related strains) differences. Further characterization of these strains by pan-genome analysis showed a total of 1,707 genes in the core genome. The accessory genome was found to contain 107 shell genes and 126 cloud genes that vary from strain to strain within the panel, and which could affect the responsiveness of these strains to vaccine treatments. A recent study characterized the pan-genome of 26 *Francisella* strains, and found the genus contained 4,053 genes, with a relatively small core genome of 692 genes (Kumar et al., 2020). However, this analysis was conducted on strains of differing species. To our knowledge, no pan-genome analysis has been conducted on strains of the same species of *F. tularensis*. Within-species variation is not well characterized in *F. tularensis*, though it is considered a clonal organism with highly conserved genomic sequence. A previous study reported an average nucleotide identity of ≥99% within subspecies of *F. tularensis* subsp. *holarctica* (Vogler et al., 2009), so the high level of genetic similarity in our study is not surprising. Strains in our panel are derived from a variety of hosts, geographical locations, and dates; therefore, the level of genomic variation we observe is representative of *F. tularensis*.

### Exclusion of FRAN031 and FRAN037 From the Vaccine-Testing Panel

Both the FRAN031 (Scherm) and FRAN037 (Coll) clinical isolate strains were described as being highly virulent (Downs et al., 1947a), and the genomic sequence for FRAN031 was previously reported in the literature (Pandya et al., 2009; Johnson et al., 2015). Based upon these previous studies, the absence of the FPI in FRAN031 and mutations in the FPI regions of FRAN037, which most likely lead to its attenuation, were unexpected observations in our current study. From the studies we have performed in a mouse model of infection, FRAN031 showed no virulence defect despite missing one of the pathogenicity islands (PAI) (Table 4). Granted, *F. novicida* also contains only one PAI and still highly virulent for mice (Biot et al., 2020).

FRAN037 was the only isolate in our panel that showed attenuation in both murine macrophages and intranasal mouse infection models. Several observations about the mutations identified in the FPI of FRAN037 may explain this attenuation. Firstly, FRAN037 harbored two unique frameshift mutations in the *pdpB1* gene. PdpB has been designated a homolog of IcmF, a known component of the type VI secretion system (T6SS). Multiple studies have demonstrated PdpB localizes to the inner membrane and is required for intracellular growth, supporting its role as a structural T6SS protein (Tempel et al., 2006; de Bruin et al., 2007, 2011; Schmerk et al., 2009b). Since PdpB1 is known to be a structural type VI secretion system protein, located on the inner membrane, a non-functional variant could potentially render the entire multi-subunit needle complex non-functional. Expression of a second mutated variant could be more deleterious to the cell than having a single functional variant. Additionally, FRAN037 contained an in-frame deletion of the first 42 amino acids of the IglA2 protein, also a unique mutation in our panel. IglA is also a structural component of the T6SS, interacting with IglB to form the contractile sheath of the needle complex. We hypothesize that the mutations found in one or both of these structural proteins, IglA and PdpB, may lead to a dominant negative effect and cause the attenuation observed in FRAN037. As such, this strain will not be included as part of the diversity panel for further testing. Likewise, the lack of the FPI in FRAN031 was unexpected. Therefore, we will also not be including this strain as part of our formal panel strain for further testing.

The exact reasons for the occurrence of the FPI mutations identified in these historical strains remains to be determined. For the purpose of this study, we attempted to use isolates as close to the original isolate as available in the USAMRIID repository (Table 1). Based upon previous literature, Coll (FRAN037) should have been a fully virulent strain in mice (Downs and Woodward, 1949). However, in our tularemia intranasal challenge model in BALB/c mice, FRAN037 was completely attenuated at the doses used. The question remains if previous handling of this isolate prior to preparation for long-term storage lead to the mutations and attenuation or if these genetic mutations were already present from original 1945 isolate. Previous mouse virulence assays (Downs and Woodward, 1949) were performed by intraperitoneal challenge versus intranasal challenge as done here. In addition, it is unclear what mouse strain was used for these previous experiments. Perhaps these differences in the virulence characterization of FRAN037 could lead to these discrepancies in the mouse LD_50_ studies and these genetic mutations in FRAN037 have always been present. The same questions remain for FRAN031 and loss of the FPI. We cannot rule out if the FPI was absent from the genome since its original isolation (1944) or this occurred at some point once prior to preparation for long term storage.

### Variation in the FPI Region

The 1958 version of Schu S4, FRAN249, harbored a frameshift mutation in the *pdpA1* gene, the first gene encoded by the FPI. Studies by Schmerk et al. (2009a, b) showed that *pdpA* of *F. novicida* was necessary for virulence in mice, replication in murine macrophages, and involved in interruption of host cell signaling. However, PdpA is not believed to be a structural component of the T6SS, since it bears no homology to structural T6SS proteins from other bacteria, and the exact biological role of PdpA is not well defined. In our study, FRAN249 showed no virulence difference in the mouse intranasal infection model; however, a functional *pdpA* copy is present on the second FPI which could compensate for a non-functional *pdpA*. Indeed, the function of many of the FPI proteins have been defined using *F. novicida*, which contains only a single FPI. Given that all of our strains, except FRAN037, retained virulence in the mouse model of intranasal infection, a simple explanation could be that a functional copy of the gene was supplied on the duplicated pathogenicity island.

Other mutations in the FPI have been previously reported in a comparative genomics study between the Schu S4 strain and WY96, representing type A1 and type A2 strains, respectively (Beckstrom-Sternberg et al., 2007). Of the 60 polymorphisms identified across both FPI regions, mutations resulting in premature stops occurred only in *pdpC*, *pdpD*, and several hypothetical proteins, while the *pdpA*, *pdpB*, and *iglABCD* genes remained intact (Beckstrom-Sternberg et al., 2007). Our study also identified frameshift mutations causing premature stops in *pdpC* and *pdpD*, indicating these genes are not as crucial for virulence. Our study identified additional mutations causing truncated variants of *pdpB1* and *iglA2*; however, they were only found in FRAN037, which was completely attenuated, whereas WY96 has been described as a virulent type A isolate. Notably, this study found no SNPs in *iglA* and determined *pdpB* to be under purifying selection, further supporting their importance in virulence and survival of *Francisella*. One unusual observation from our study was the presence of two identical mutations in FPI genes of two different panel strains, FRAN244 and FRAN251. Although these strains differ in geographical origin, clinical source, and date of isolation (1941 vs. 2017), they contain the same mutations: 1647delT in *iglF1* and 2045delA in *pdpC1*.

Notably, FRAN251 is the only isolate in our panel that is presumed to contain two non-functional copies of an FPI gene. Both copies of *pdpC* contain a single bp deletion causing a frameshift and premature stop codon. A previous study assessed the effect of loss of *pdpC* in LVS on expression of the surrounding FPI genes and showed that membrane integrity was still maintained in the mutant, but it was unable to escape the phagosome and was significantly attenuated during intradermal infection of mice. Interestingly the *pdpC* LVS mutant exhibited a cytopathogenic response, due to its fragmentation of the phagosomal membrane and subsequent triggering of the inflammasome (Lindgren et al., 2013); this study noted that this is very different from the effect of an *iglC* mutant which does not show this hyper-cytotoxicity.

Interestingly, the *anmK* genes of FRAN256 differed from the rest of the panel within both FPI regions. It has been previously reported that *anmK* within *F. novicida* (also referred to as *pmcA*), encoding a 371 amino acid chaperone-like protein, is divided into two ORFs in Schu S4 (Nano and Schmerk, 2007). Similarly, all the type A isolates in our panel, with the exception of FRAN256, contained two ORFs, *anmK1* and *anmK2*. FRAN256 harbored a single ORF for *anmK* in FPI-1, while *anmK1* harbored a 246 bp insertion, and *anmK2* harbored a deletion causing a truncated variant. Type B strains were previously reported to contain a large deletion in the *anmK* and *pdpD* genes (Nano et al., 2004); however, FRAN255 harbored no such deletion and showed similar ORFs in this region as compared to the type A variants.

The presence of the observed SNPs within the FPIs of these strains was unexpected since this region encodes for proteins making up the Type VI secretion system and shown to be essential for virulence. The PAI is duplicated in all subspecies of *F. tularensis*, except for *F. novicida* (Nano et al., 2004; Larsson et al., 2009). The exact role for the duplication of the PAI duplication remains to be determined. The G + C content of the FPI in contrast to the rest of the *Francisella* genome suggests it was acquired via horizontal gene transfer. Evidence suggests that the redundancy of the FPI compensates for the loss of some of the loci in this region (Bröms et al., 2010). The results of our current study would support this idea as we found SNPs in the FPI of five of the panel strains, but they retained full virulence in the pneumonic murine tularemia model. The exception to this is the FRAN037/Coll strain in which mutations occurred in genes encoding for structural proteins, *pdpB* and *iglA*. However, a previous study suggests (at least for LVS) both copies of *iglABCD* are necessary for optimal virulence in mice (Su et al., 2007).

### Variation in Other Genes of Interest

The UpSet plot analysis of the *F. tularensis* panel showed a large number of unique genes within the FRAN254, FRAN255, and FRAN256 strains as compared to the rest of the panel. Two of these genes corresponded to IS5 family transposase elements, ISFtu1 an ISFtu2, which were duplicated at differing frequencies across strains. Interestingly, the FRAN256 genome contained 26 copies of this mobile element while other panel strains contained up to 16 copies. These copies occurred in the same locations as those identified in the Schu S4 strain, with the exception that many of those elements were duplicated at those loci in the FRAN256 strain. Conversely, we observed that a second mobile element, ISFtu1, lacked additional copies in FRAN256 that were observed in other strains. This element was repeated 46–51 times in the genome of our panel strains, but only occurred nine times in FRAN256. Transposase activity often leads to genome rearrangements and gene deletions in bacteria, and it has been proposed that expansion of IS elements occurs as free-living bacteria transition to an intracellular lifestyle, where many of the genes are non-essential in the nutrient-rich host environment (Moran and Plague, 2004). The difference in IS element copy number in FRAN256 could indicate this strain is at a different evolutionary stage than the other strains included in our panel.

In our UpSet plot, a single gene group was found to be shared between FRAN254, FRAN255, and FRAN256. Further investigation revealed three copies of this gene in the *Francisella* genome, and that the encoded protein harbors a glycosyltransferase domain containing a DXD motif (PFAM04488). Interestingly, FRAN254, FRAN255, and FRAN256 harbor one functional copy of this gene, and two copies containing frameshift mutations. In the *F. tularensis* Schu S4 genome, these genes correspond to locus tags FTT_0354, FTT_0378c, and FTT_1263c. A protein databank search of this sequence also showed that it harbors 54% sequence similarity, although only 11% query cover, to the glucosyltransferase domain (GTD) of the *Clostridium difficile* toxin A (TcdA). Upon binding of the TcdA toxin to host cells, the GTD portion of the toxin is released and acts upon Rho GTPases in the cytoplasm to inactivate them via glucosylation, which triggers downstream cytopathic and cytotoxic effects (Di Bella et al., 2016). It is possible this domain may serve a similar function in the pathogenesis of *Francisella*. A previous study identified up to four homologs of this protein in *F. tularensis* and *F. holarctica* strains, while *F. novicida* lacked a homolog of this glycosyltransferase (Barabote et al., 2009). More recently, Nau et al. (2019) demonstrated that a homolog of this transglycosylase in LVS, designated GdcA, was able to inhibit NF-KB signaling in immune cells.

### Phenotypic Differences Observed in the *F. tularensis* Panel

Growth of the strains in our *F. tularensis* panel was assessed in both the nutrient deplete BHI supplemented with 1% Isovitalex or the nutrient replete CDM. Interestingly, both Schu S4 strains (FRAN244 and FRAN249) grew significantly better in BHI than the other *F. tularensis* strains tested. The exact reason for enhanced growth of the Schu S4 in supplemented BHI as compared to the other strains tested here remains to be determined. Previous work showed that growth of *Francisella* in BHI broth elicited protein expression profiles similar to that of *Francisella* grown in macrophages, whereas growth in CDM and Mueller Hinton broth yielded different profiles (Hazlett et al., 2008; Holland et al., 2017). Therefore, growth differences between strains in BHI could indicate growth defects in macrophages. However, all of these strains replicated similarly in macrophages, except for FRAN037 that was decreased in CFU recovery, although not significant. Interestingly, this strain was the only one in our panel to show a growth defect in the nutrient replete CDM. It is possible the mutated FPI genes in FRAN037 contribute to this phenotype though further investigation is needed.

We also examined the LPS and capsule profiles of the panel strains using monoclonal antibodies directed against the O-antigens of these respective structures. In general when using these antibodies for characterization, no differences in the profiles were noted between the strains other than intensity differences in the banding pattern for FRAN255 at the higher molecular weight range for both the LPS and capsule. However, this does not rule out other possible differences in the LPS between strains with additional characterization. From the genomic analysis, several of the genes mutated in the *F. tularensis* panel are involved in LPS biosynthesis and transport. For example, FRAN254 contains a frameshift within the *lpxL2* gene, encoding the lipid A lauryoyltransferase. The closest relative of LpxL2 is LpxM from *Acinetobacter baumannii* (Dovala et al., 2016). LpxL and LpxM are known to share significant sequence homology and functional similarity (Six et al., 2008). In the *E. coli* LPS biosynthesis pathway, LpxL catalyzes the transfer of laurate to the Kdo_2_-lipid IV_*A*_, which then becomes the substrate for myristoylation by LpxM, the final enzyme in the pathway (Six et al., 2008). It has been demonstrated that deletion of *lpxL* and *lpxM* homologs in *Neisseria meningitidis* affects the resultant structure of the LPS as well as its toxicity and adjuvant activity (van der Ley et al., 2001). Interestingly, an additional *lpxL* gene encoding a smaller variant of 299 amino acids was found to be identical among all strains in our panel, indicating potential redundancy of function. These LpxL variants in *Francisella* have been previously characterized, and *lpxL* was shown to be necessary for LPS acylation, while deletion of *lpxL2* did not appear to affect the lipid A species produced (McLendon et al., 2007).

In addition to the *lpxL* mutation, we found two mutations in *lpt* genes of two panel strains. The Lpt complex is a superstructure made of the proteins LptA-LptG and is required for LPS transport across the bacterial membrane (Wu et al., 2006). FRAN256 contained a mutation in *lptA* and FRAN255 was found to have multiple mutations in *lptD*. In *E. coli*, LptA functions to bind and transport LPS across the periplasm to the outer membrane (Schultz et al., 2017), although this protein is not well-studied in *Francisella*. The mutation identified in FRAN256 results in a frameshift and truncated protein product of 139 amino acids, in contrast to the full-length 277 amino acid variant. While no obvious LPS or capsule defect was observed in whole cell extracts of FRAN256 in this study, further investigation is required to determine if there are any subtle defects we were not able to detect by the methods employed here. The *lptD* gene, also known as *ostA*, encoding for the organic solvent tolerance protein, has two variants in *Francisella*- *ostA1* and *ostA2*. A previous study showed that deletion of *ostA2* in *F. novicida* resulted in the inability to attach and form biofilms compared to the wild-type strain (Margolis et al., 2010). This gene corresponds to *ostA2*, as evidenced by its location in the genome and sequence similarity, although it has unique features compared to the *lptD* gene from the type A isolates, including a deletion of the first 522 bp, a 3 bp in-frame deletion toward the middle of the gene, and a 180 bp extension at the end of the gene. Perhaps the slight variation observed at the higher molecular weight range of the LPS and capsule profile for FRAN255 could be contributable to this alteration for the *lptD* gene.

### Future Studies With the *F. tularensis* Challenge Panel

We are further developing this challenge panel to confirm the protective efficacy of future vaccines against pneumonic tularemia using an array of diverse *F. tularensis* strains beyond the prototype Schu S4. Due to the loss of the duplicated FPI of FRAN031 and the attenuation of FRAN037, these two strains have been eliminated from the panel and additional *F. tularensis* strains are being explored for inclusion. Preferential consideration is being given to type B strains to expand their representation as part of the panel. In addition, a recent publication has further characterized the MA00-2987 strain, a type A strain isolated from a human in 2000 from Massachusetts, U.S. in the Fischer 344 rat model (Lovchik et al., 2021). We are currently considering the addition of this strain to the panel for future testing.

Indeed, the strains making up this panel will be evolving as additional *F. tularensis* isolates of interest become available to our collection. For the strains already included in the panel, we have produced well-characterized challenge material and begun to assess virulence of these strains in a rat model of inhalational tularemia, a more appropriate challenge model for identifying virulence differences. The current *F. tularensis* panel and the Fischer rat inhalational tularemia model provides a framework to begin to assess the efficacy of vaccine candidates against a variety of strains beyond the prototypical Schu S4 strain. For those vaccine candidates shown to be protective in the rat model, they could be transitioned for additional efficacy testing in the non-human primate pneumonic tularemia challenge model.

## Data Availability Statement

The datasets presented in this study can be found in online repositories. The names of the repository/repositories and accession number(s) can be found in the article/Supplementary Material.

## Ethics Statement

The animal study was reviewed and approved by The United States Army of Medical Research Institute of Infectious Diseases Institutional Animal Care and Use Committee (USAMRIID IACUC).

## Author Contributions

BB, JR, KM, DR, and JB contributed conception and design of the study and wrote the manuscript. BB, JR, KM, CK, RT, AL, KC, JS, AR, CC, DR, and JB participated in the experimentation and acquisition of data. BB, JR, KM, DF, DR, and JB were involved in the analysis or interpretation of data for the work. All the authors contributed to manuscript revision, read and approved the submitted version.

## Author Disclaimer

Opinions, interpretations, conclusions, and recommendations are those of the authors and are not necessarily endorsed by the U.S. Army.

## Conflict of Interest

The authors declare that the research was conducted in the absence of any commercial or financial relationships that could be construed as a potential conflict of interest.

## Publisher’s Note

All claims expressed in this article are solely those of the authors and do not necessarily represent those of their affiliated organizations, or those of the publisher, the editors and the reviewers. Any product that may be evaluated in this article, or claim that may be made by its manufacturer, is not guaranteed or endorsed by the publisher.
