# Supplementary material for: Development, Phenotypic Characterization and Genomic Analysis of a Francisella tularensis Panel for Tularemia Vaccine Testing
Source: Front Microbiol. 2021 Aug 11;12:725776. doi: 10.3389/fmicb.2021.725776 (PMC8386241; doi:10.3389/fmicb.2021.725776)
Supplement: Supplementary file 1 [file Presentation_1.pdf]

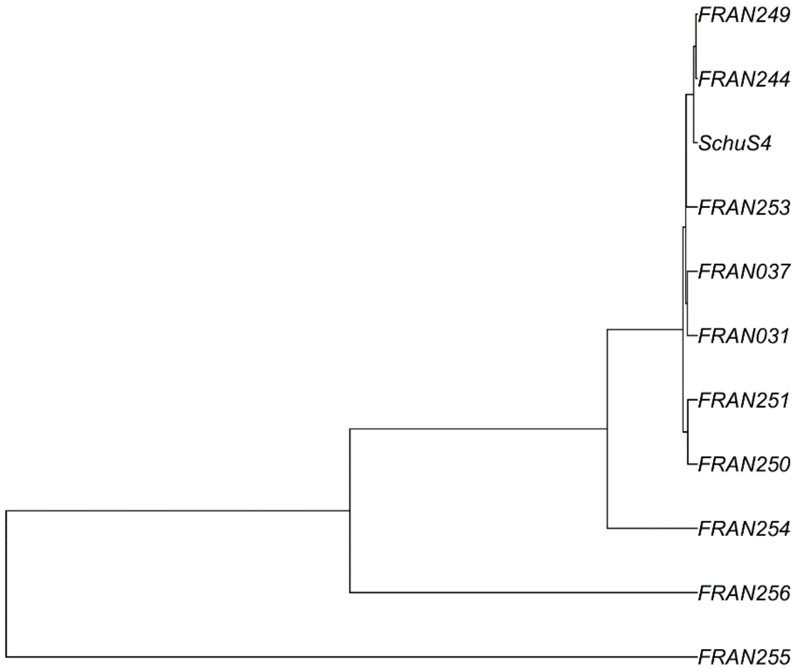

	FRAN249	*FRAN244*	*SchuS4*	*FRAN253*	*FRAN037*	*FRAN031*	*FRAN251*	*FRAN250*	*FRAN254*	*FRAN256*	*FRAN255*
FRAN249	0	1.23E-05	3.43E-05	0.000105	9.41E-05	9.37E-05	0.00012	0.000133	0.000847	0.003145	0.006395
FRAN244	1.23E-05	0	3.49E-05	9.48E-05	0.000106	0.000122	0.000124	0.000134	0.000846	0.003146	0.00637
SchuS4	3.43E-05	3.49E-05	0	0.000117	0.000126	0.000117	0.00015	0.000157	0.00088	0.003171	0.006431
FRAN253	0.000105	9.48E-05	0.000117	0	0.000115	9.67E-05	0.000142	8.93E-05	0.000811	0.003095	0.006435
FRAN037	9.41E-05	0.000106	0.000126	0.000115	0	9.32E-05	0.000164	0.000129	0.000796	0.003123	0.006405
FRAN031	9.37E-05	0.000122	0.000117	9.67E-05	9.32E-05	0	0.000118	0.000141	0.000864	0.003202	0.006403
FRAN251	0.00012	0.000124	0.00015	0.000142	0.000164	0.000118	0	8.91E-05	0.000802	0.003202	0.006436
FRAN250	0.000133	0.000134	0.000157	8.93E-05	0.000129	0.000141	8.91E-05	0	0.000869	0.00318	0.006474
FRAN254	0.000847	0.000846	0.00088	0.000811	0.000796	0.000864	0.000802	0.000869	0	0.003883	0.00709
FRAN256	0.003145	0.003146	0.003171	0.003095	0.003123	0.003202	0.003202	0.00318	0.003883	0	0.005978
FRAN255	0.006395	0.00637	0.006431	0.006435	0.006405	0.006403	0.006436	0.006474	0.00709	0.005978	0

Supplementary Figure 1. Percentage difference between each strain based on average nucleotide identity calculated by mutual BLAST. Green indicates a greater distance between strains, red indicates greater similarity.

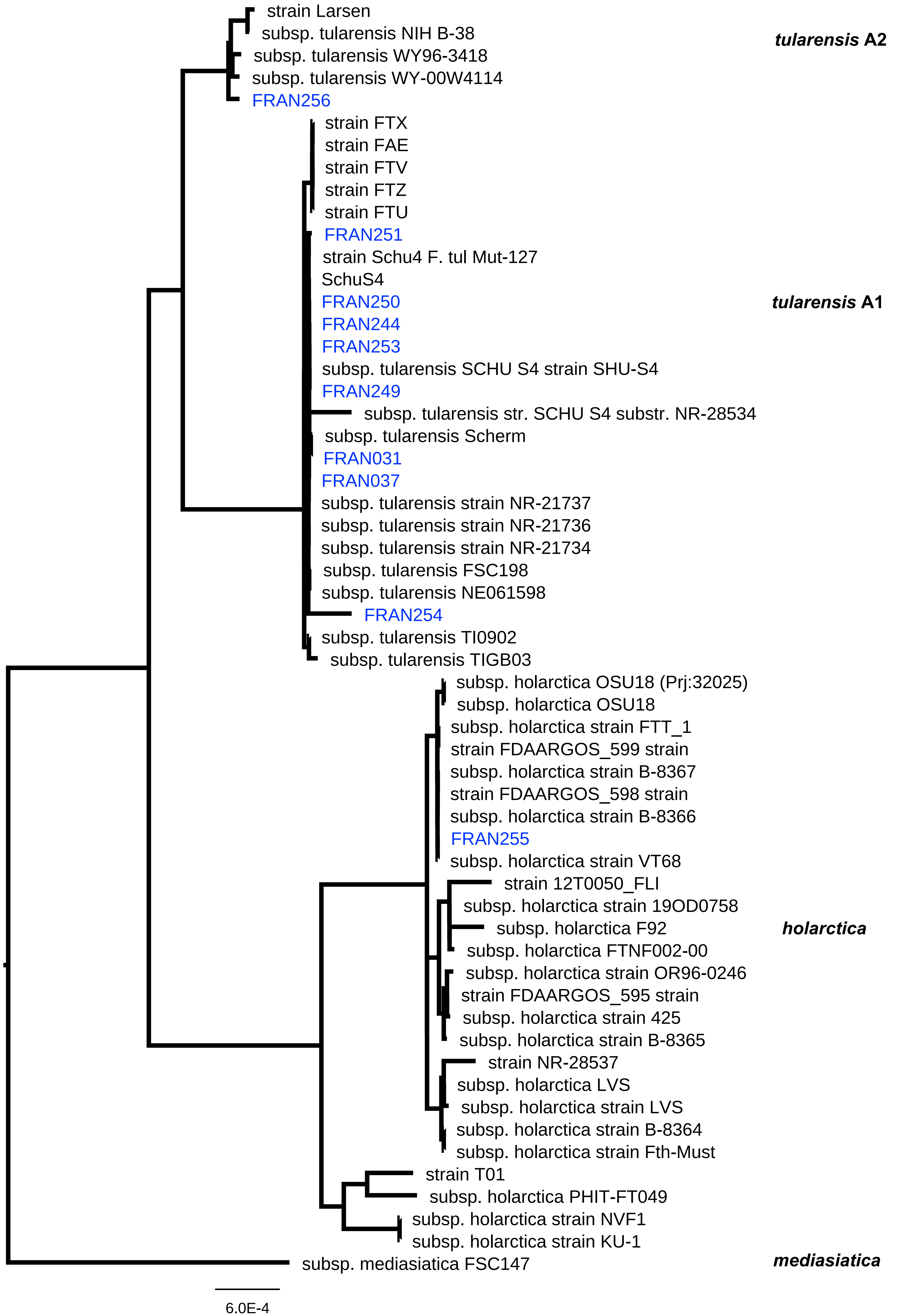

strain Larsen
subsp. tularensis NIH B-38
subsp. tularensis WY96-3418
subsp. tularensis WY-00W4114
FRAN256
tularensis A2
strain FTX
strain FAE
strain FTV
strain FTZ
strain FTU
FRAN251
strain Schu4 F. tul Mut-127
SchuS4
FRAN250
FRAN244
FRAN253
subsp. tularensis SCHU S4 strain SHU-S4
FRAN249
subsp. tularensis str. SCHU S4 substr. NR-28534
subsp. tularensis Scherm
FRAN031
FRAN037
subsp. tularensis strain NR-21737
subsp. tularensis strain NR-21736
subsp. tularensis strain NR-21734
subsp. tularensis FSC198
subsp. tularensis NE061598
FRAN254
subsp. tularensis TI0902
subsp. tularensis TIGB03
tularensis A1
subsp. holarctica OSU18 (Prj:32025)
subsp. holarctica OSU18
subsp. holarctica strain FTT_1
strain FDAARGOS_599 strain
subsp. holarctica strain B-8367
strain FDAARGOS_598 strain
subsp. holarctica strain B-8366
FRAN255
subsp. holarctica strain VT68
strain 12T0050_FLI
subsp. holarctica strain 19OD0758
subsp. holarctica F92
subsp. holarctica FTNF002-00
subsp. holarctica strain OR96-0246
strain FDAARGOS_595 strain
subsp. holarctica strain 425
subsp. holarctica strain B-8365
strain NR-28537
subsp. holarctica LVS
subsp. holarctica strain LVS
subsp. holarctica strain B-8364
subsp. holarctica strain Fth-Must
strain T01
subsp. holarctica PHIT-FT049
subsp. holarctica strain NVF1
subsp. holarctica strain KU-1
holarctica
subsp. mediasiatica FSC147
mediasiatica
6.0E-4

Supplementary Figure 2. Phylogeny of *F. tularensis* strains with completed genomes. The tree is comprised of *F. tularensis* subsp. *holarctica*, *tularensis* and *mediasiatica* strains that have complete genomes, including the newly sequenced strains in our panel, indicated in blue. The PATRIC phylogenetic tree service was used to generate a RaxML tree based on 100 conserved genes found within each strain. The genomes fall within three main clades corresponding to *holarctica*, *tularensis* type A2 and *tularensis* type A1. Phylogenetic distance, measured by substitutions/site, is indicated on the x-axis.

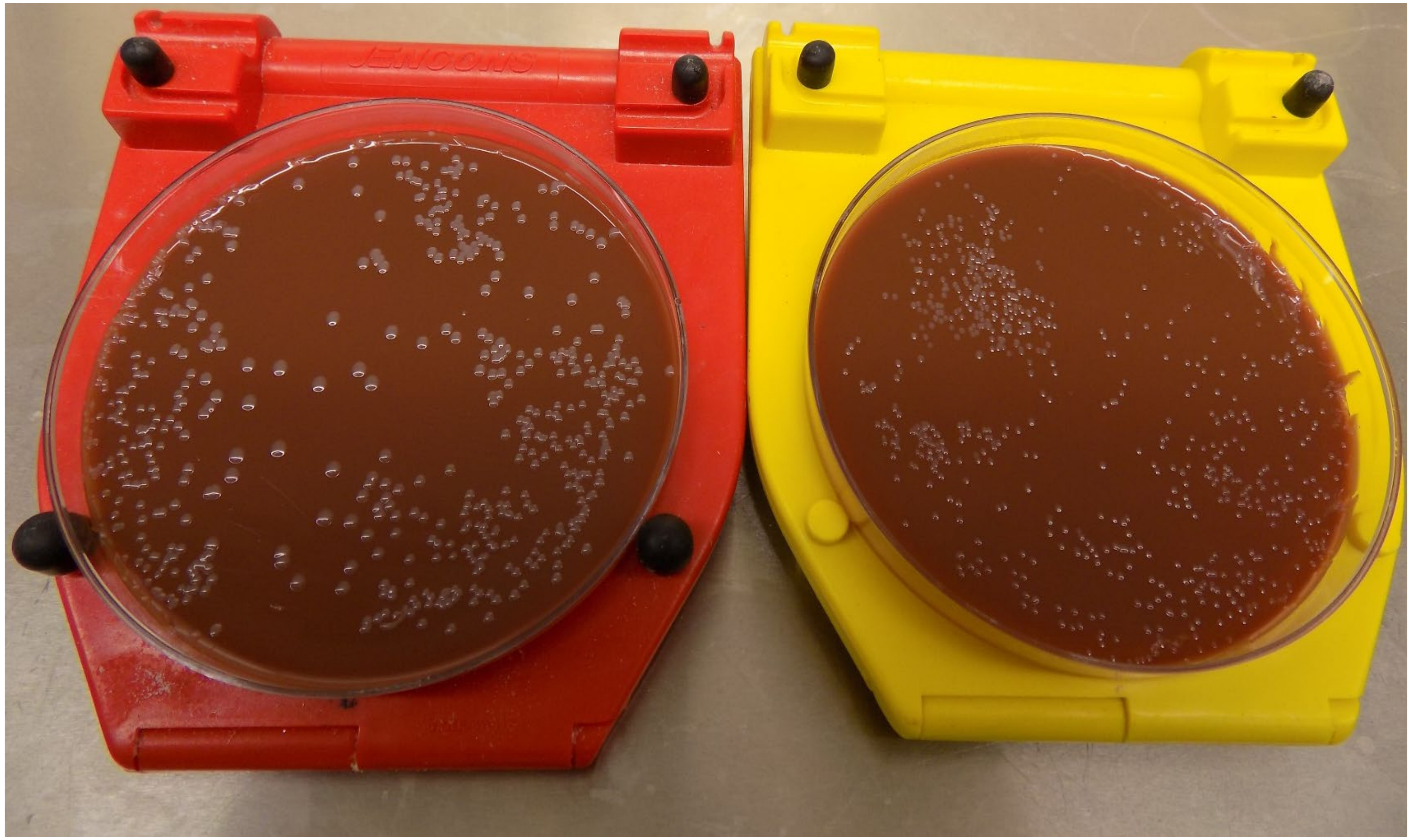

Supplementary Figure 3. Colony appearance of *F. tularensis* Schu S4 (left) and FRAN255 (right) after 2 days of growth on Remel chocolate agar plates.
