# Supplementary material for: Development, Phenotypic Characterization and Genomic Analysis of a Francisella tularensis Panel for Tularemia Vaccine Testing
Source: Front Microbiol. 2021 Aug 11;12:725776. doi: 10.3389/fmicb.2021.725776 (PMC8386241; doi:10.3389/fmicb.2021.725776)
Supplement: Supplementary file 2 [file Presentation_2.PDF]

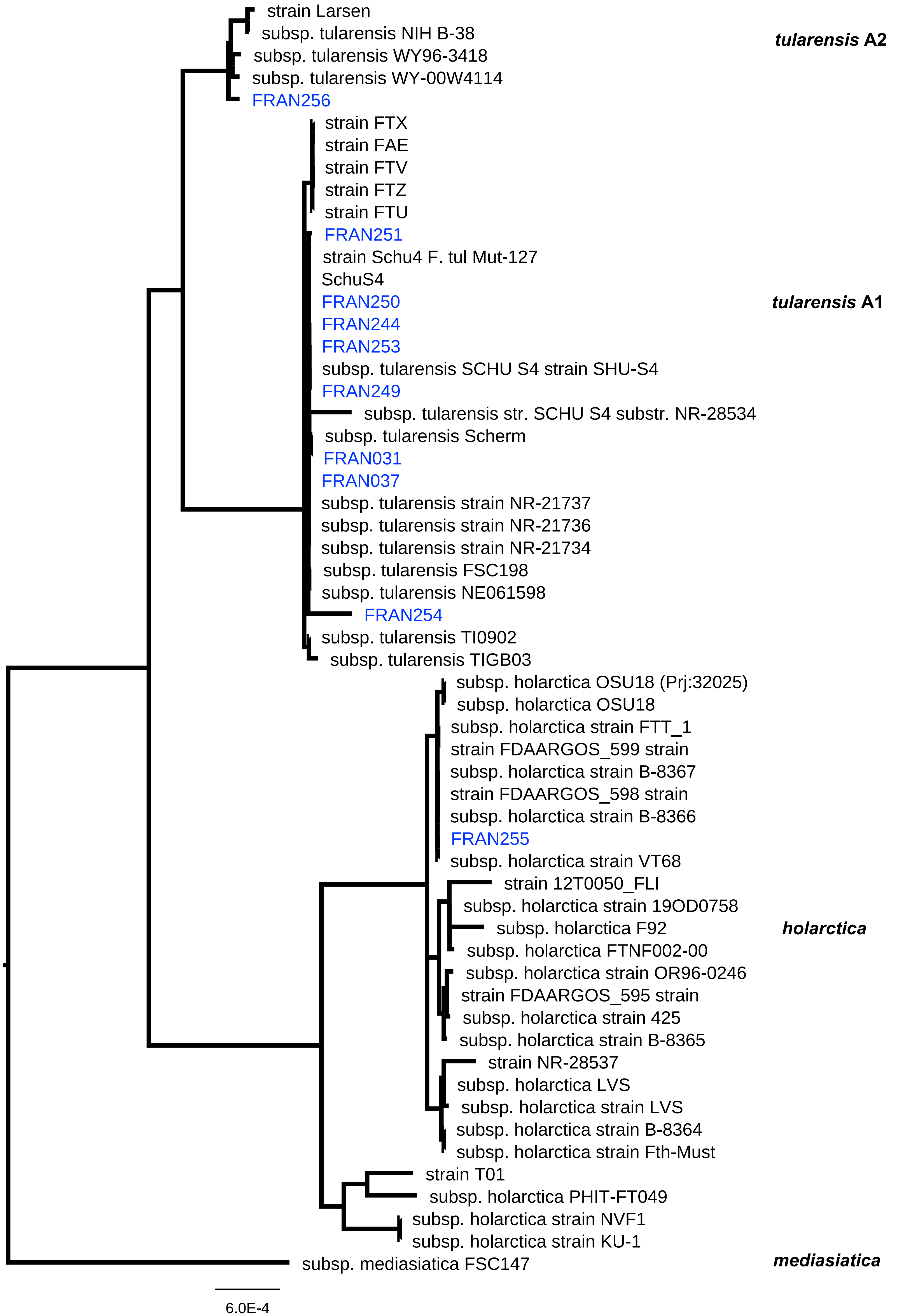

strain Larsen
subsp. tularensis NIH B-38
subsp. tularensis WY96-3418
subsp. tularensis WY-00W4114
FRAN256
tularensis A2
strain FTX
strain FAE
strain FTV
strain FTZ
strain FTU
FRAN251
strain Schu4 F. tul Mut-127
SchuS4
FRAN250
FRAN244
FRAN253
subsp. tularensis SCHU S4 strain SHU-S4
FRAN249
subsp. tularensis str. SCHU S4 substr. NR-28534
subsp. tularensis Scherm
FRAN031
FRAN037
subsp. tularensis strain NR-21737
subsp. tularensis strain NR-21736
subsp. tularensis strain NR-21734
subsp. tularensis FSC198
subsp. tularensis NE061598
FRAN254
subsp. tularensis TI0902
subsp. tularensis TIGB03
tularensis A1
subsp. holarctica OSU18 (Prj:32025)
subsp. holarctica OSU18
subsp. holarctica strain FTT_1
strain FDAARGOS_599 strain
subsp. holarctica strain B-8367
strain FDAARGOS_598 strain
subsp. holarctica strain B-8366
FRAN255
subsp. holarctica strain VT68
strain 12T0050_FLI
subsp. holarctica strain 19OD0758
subsp. holarctica F92
subsp. holarctica FTNF002-00
subsp. holarctica strain OR96-0246
strain FDAARGOS_595 strain
subsp. holarctica strain 425
subsp. holarctica strain B-8365
strain NR-28537
subsp. holarctica LVS
subsp. holarctica strain LVS
subsp. holarctica strain B-8364
subsp. holarctica strain Fth-Must
strain T01
subsp. holarctica PHIT-FT049
subsp. holarctica strain NVF1
subsp. holarctica strain KU-1
holarctica
subsp. mediasiatica FSC147
mediasiatica
6.0E-4

Supplementary Figure S2. Phylogeny of *F. tularensis* strains with completed genomes. The tree is comprised of *F. tularensis* subsp. *holarctica*, *tularensis* and *mediasiatica* strains that have complete genomes, including the newly sequenced strains in our panel, indicated in blue. The PATRIC phylogenetic tree service was used to generate a RaxML tree based on 100 conserved genes found within each strain. The genomes fall within three main clades corresponding to *holarctica*, *tularensis* type A2 and *tularensis* type A1. Phylogenetic distance, measured by substitutions/site, is indicated on the x-axis.
